# Impact of synthesis methods on the functionality of antibody-conjugated gold nanoparticles for targeted therapy†

**DOI:** 10.1039/d4na00134f

**Published:** 2024-09-04

**Authors:** Adi Anaki, Chen Tzror-Azankot, Menachem Motiei, Tamar Sadan, Rachela Popovtzer

**Affiliations:** a Faculty of Engineering and the Institute of Nanotechnology and Advanced Materials, Bar-Ilan University Ramat Gan 5290002 Israel rachela.popovtzer@biu.ac.il

## Abstract

Gold nanoparticles (GNPs) are emerging as promising modular platforms for antibody-based cancer therapeutics. Their unique physiochemical properties enable efficient binding of multiple antibodies upon a single particle, thereby enhancing therapeutic potential. However, the effect of widely used synthesis techniques on the characteristics and functionality of antibody-GNP platforms has yet to be fully understood. Here, we investigated the effect of key synthesis approaches, namely, covalent binding and physical adsorption, on the properties and anti-cancer functionality of antibody-coated GNPs. By carefully manipulating synthesis variables, including antibody mass in reaction and linker compositions, we revealed a direct impact of these synthesis methods on antibody binding efficiency and anti-cancer functionality. We found that covalent binding of antibodies to GNPs generated a platform with increased cancer cell killing functionality as compared to the adsorption approach. Additionally, a higher antibody mass in the synthesis reaction and a higher polyethylene glycol linker ratio upon covalently bound antibody-GNPs led to increased cell death. Our findings emphasize the critical role of synthesis strategies in determining the functionality of targeted GNPs for effective cancer therapy.

## Abstract 2

This study investigated the impact of key synthesis approaches, namely, covalent binding and physical adsorption, on the properties and anti-cancer functionality of antibody-coated gold nanoparticles.

## Graphical abstract

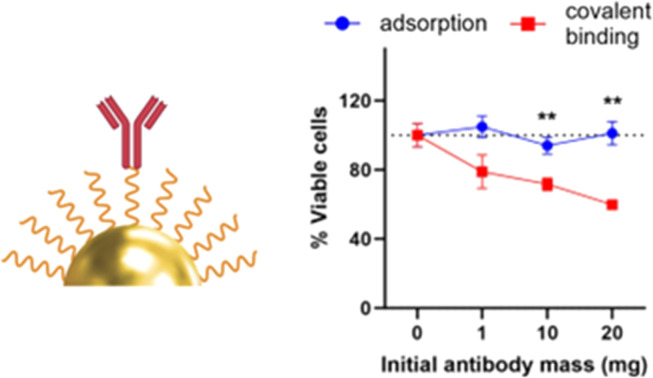

## Introduction

Antibody-based therapy for cancer is currently one of the most prominent strategies for treating patients with hematological malignancies and solid tumors. Various therapeutic antibody formats, including monoclonal antibodies as well as new formats such as antibody fragments, bispecific antibodies, and antibody–drug conjugates, show promising clinical success in cancer treatment. Antibody-mediated killing of tumor cells can result from the direct action of the antibody on a tumor cell antigen, from the antibody's specific effects on tumor vasculature and stroma, *via* immune-mediated cell killing mechanisms, or by payload delivery. Nevertheless, these antibody formats have various challenges: the biodistribution and pharmacokinetics of these different therapeutic antibody classes generally result in a limited percentage of injected antibodies that ultimately localize within a solid tumor.^1–3^ The less-than-optimal pharmacokinetic profiles of such therapeutic antibodies can necessitate frequent administration to achieve desired outcomes.^4–6^ Such antibodies also have a limited ability to penetrate and distribute within the tumour, which restricts the full extent of treatment efficacy.^7–11^ In addition, merging two or more antibodies into a single entity, to create bi- and multi-specific antibodies, can generate reduced affinity and specificity of the new entity towards its tumor cell target.

Recent advances in nanotechnology offer new avenues with significant potential to revolutionize antibody-based therapies for cancer.^4,12^ Combining the precision of antibodies for targeting of cancer-associated antigens, together with the distinct attributes of nanoparticles, creates new and enhanced targeted delivery systems that can improve therapeutic efficacy.^12,13^ Nanoparticles offer a platform to which biomolecules can be simultaneously linked while remaining minimally affected, that is, with retention of their original biological properties.^13^ Furthermore, nanoparticles have different whole-body biodistribution patterns as compared to free antibodies, which can decrease the exposure of healthy tissues to therapy and thus reduce side effects.^14^ Gold nanoparticles (GNPs), in particular, have garnered significant attention due to their unique physicochemical properties and potential biomedical applications.^15–21^ GNPs are biocompatible and have tunable and controllable physiochemical features.^16,18,22^ GNPs' surface properties enable straightforward conjugation of versatile payload combinations to the same particle, and their high surface area-to-volume ratio allows delivery of large payloads, including antibodies.^8^ Moreover, while antibody–drug conjugates and bi- and multi-specific formats are difficult to engineer using traditional techniques, multifunctional nanoparticles, including antibody-conjugated GNPs, can be easily and precisely engineered using existing chemistries.^12^ Addition of coatings such as polyethylene glycol (PEG) has been shown to improve nanoparticle stability, solubility, and biocompatibility,^23,24^ protect nanoparticles from detection by the immune system and prolong circulation time.^25–27^ Various nanoparticles, including GNPs, can enhance the delivery and accumulation of antibodies within tumors as compared to free antibodies,^28,29^ primarily due to the enhanced permeability and retention (EPR) effect, characterized by leaky tumor vasculature and impaired lymphatic drainage.^14^

To date, various design and synthetic approaches of antibody-GNP platforms (Ab-GNP) have been proposed, each which can directly impact the platform's physicochemical features and anti-cancer functionality.^30–33^ The two widely used synthetic approaches for binding antibodies upon GNPs are physical adsorption onto the particle surface, providing a simple and straightforward method for attachment through electrostatic interactions between the antibody and the nanoparticle surface,^34–38^ and covalent binding that provides conjugation by chemical coupling between functional groups of the surface linker (such as PEG) and the antibodies.^15,31,32,39^ Despite the prevalence of these synthesis approaches, a comprehensive understanding of their impacts on the properties and functionality of Ab-GNP platforms remains unclear.

In the present study, we investigated the direct impact of these two synthesis approaches on Ab-GNP features. By carefully manipulating key synthesis variables, including antibody mass in the initial reaction and PEG linker compositions, we studied the outcome of the different Ab-GNP synthesis approaches, in terms of physicochemical properties, antibody coupling efficiency, and most importantly, anti-cancer cell functionality. As a model antibody for investigation of the Ab-GNP platforms, we used cetuximab, a clinically employed monoclonal antibody that binds to the extracellular domain of the epidermal growth factor receptor (EGFR), which is overexpressed in many human cancers.^40–42^ Binding of the antibody to EGFR blocks activation of multiple kinases and leads to subsequent tumor cell growth inhibition and apoptosis.^40–42^ Our results show a relationship between the different synthesis parameters and Ab-GNP functionality. The findings can contribute to the rational design of Ab-GNPs for targeted therapy, enabling more precise and effective treatments for cancer patients.

## Material and methods

### GNP synthesis

Synthesis of 20 nm spherical GNPs was carried out using sodium citrate (Sigma Aldrich) as a reducing agent, based on Enüstün and Turkevic's methodology.^43^ A 414 μL portion of 50% w/v HAuCl_4_ solution was added to 200 mL purified water (Hylabs, Rehovot, Israel), and the solution was boiled in a paraffin oil bath. Then, 4.04 mL of 10% sodium citrate solution were added, and the solution was stirred for 5 min. After being cooled to room temperature, the GNPs were coated with a PEG layer of either thiol-polyethylene-glycol (mPEG-SH, *M*_w_ ≈ 6 kDa, Sigma Aldrich) or a heterofunctional thiol-PEG-carboxylic acid (SH-PEG-COOH, *M*_w_ ≈ 5 kDa, Sigma Aldrich). The PEG types were added in excess to the nanoparticles and the solutions were stirred for 1 h at room temperature. PEG coatings were at ratios of either 0 : 100, 15 : 85, 50 : 50, or 100 : 0 of PEG-COOH : mPEG (the % weight for PEG (gr PEG/gr Au) was 6% g g^−1^, 20% g g^−1^, and 40% g g^−1^, for 15%, 50%, and 100% PEG-COOH coatings; and 48% for 100% mPEG). Following this step, the solutions were centrifuged to remove excess PEG molecules.

### Cetuximab binding

The cetuximab antibody layer (anti-EGFR, cetuximab (C225), Erbitux, Merck KGaA, Darmstadt, Germany) was bound to the PEG-coated particles *via* either physical adsorption or covalent binding. An increasing quantity of antibodies was used in each reaction (1 mg, 10 mg, or 20 mg cetuximab). The adsorption method was implemented *via* simple incubation of antibodies with the GNPs at room temperature, overnight. To create Ab-GNPs with a covalent bond, the carboxyl group of SH-PEG-COOH was bound to an antibody's amine group: carboxylic groups of PEG coating were activated for 30 min with 1-ethyl-3-(3-(dimethylamino)propyl)carbodiimide HCl (EDC) and *N*-hydroxy-sulfo-succinimide sodium salt (NHS) (Thermo Scientific, Waltham, MA, USA); after activation, the excess EDC/NHS was discarded by centrifugation and the solvent was washed and replaced with PBS (Hylabs), and stirred overnight with the antibodies (at increasing masses). Centrifugation was then performed, and the pellet (of generated Ab-GNPs) was collected as well as the supernatant of the reaction.

### Characterization of the nanoparticles

GNPs were characterized *via* transmission electron microscopy (TEM; JEM-1400, JEOL, Tokyo, Japan) to measure the size and shape of the GNPs, and then further characterization was conducted using ultraviolet-visible spectroscopy (UV-vis; UV-1650 PC; Shimadzu Corp., Kyoto, Japan), dynamic light scattering (DLS; NANO-flex, Particle Matrix, Germany) and zeta potential (ZetaSizer 3000HS; Malvern Instruments, Malvern, UK) measurements, following each level of coating. Stability (after 24 h incubation of adsorbed or covalently bound Ab-GNPs in PBS or DMEM supplemented with 10% fetal bovine serum (FBS)) and binding assays were examined using Nanodrop (NanoDrop™ 2000/2000c Spectrophotometers, Thermo Scientific, Waltham, MA, USA) and plate reader (Synergy™ H1, Agilent, Santa Clara, US), measurement of absorbance at 280 nm.

### Antibody binding yield

The supernatant of the synthesis reaction was collected after final centrifugation, for measurement of the binding yield of the antibodies to GNPs. Binding yield was measured by bicinchoninic acid (BCA (assay (Pierce Protein Assay, Merck, Darmstadt, Germany) of the antibodies remaining in the reaction supernatant after synthesis. The assay was performed according to manufacturer's instructions. Binding yield was then calculated using eqn (1) below.1
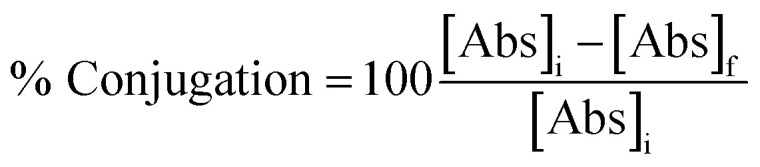
[Abs]_i_ = initial antibody mass in Ab-GNP synthesis reaction; [Abs]_f_ = final antibody mass in supernatant.

### Cell culture

Human squamous cell carcinoma A431 cells, which highly express EGFR on their cell surface,^44^ or 3T3/NIH fibroblast cell line with low EGFR expression^45^ were seeded in T75 flasks. The cells were maintained in 5 mL of Dulbecco's modified Eagle's medium (DMEM) (Sigma-Aldrich) supplemented with 10% fetal bovine serum (FBS), 2 mM l-glutamine, and 1% penicillin/streptomycin (Biological Industries.

### Methylene blue assay for study of Ab-GNP anti-cancer functionality

A431 cells or 3T3/NIH cells were counted before treatment by hemocytometer and seeded in equal amounts in normal growth conditions. After 24 h, the different types of antibody-conjugated GNPs were added to the cells (three samples for each type) at a final total gold concentration of 50 μg mL^−1^. In addition, free antibodies used as control at doses equivalent to bound antibodies upon Ab-GNPs (at 10 mg and 20 mg initial antibody mass): 18 nM, 180 nM and 320 nM. The cells were then incubated with each Ab-GNP type in normal culture conditions for 72 h (as shown in ref. 46–52). The cell viability testing was performed after washing dead cells twice with PBS and fixation with cold methanol at −20 °C for 6 minutes. Cells were then stained with 1% (% m/v) methylene blue hydrate (Sigma Aldrich) for 1 h at room temperature followed by washing twice with PBS and dissolution of stained cells with 0.1 N of HCl (Carlo Erba). Dissolving was performed at 37 °C for 30 min and absorption was read at 620 nm using a plate reader.

### Statistical analyses

Statistical significance was determined by Student's *t*-test. For multiple samples, one-way ANOVA was performed with Tukey or Dunnett post hoc corrections. *P* values below 0.05 were considered statistically significant unless stated otherwise. Statistical analyses were performed in GraphPad Prism 10 (GraphPad Software, Inc).

## Results

### Synthesis and characterization of Abs-GNPs

**Fig. 1:**
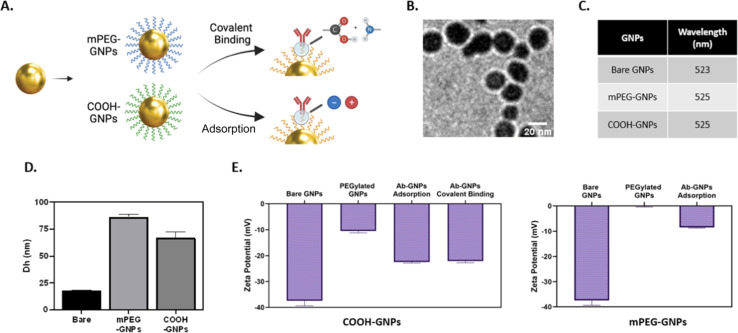
Ab-GNP synthesis and characterization. (A) Scheme of synthesis strategies of Ab-GNPs. GNPs (20 nm) were coated with either PEG-COOH or mPEG, then antibodies were bound upon GNPs using covalent binding or adsorption. (B) TEM image of the nanoparticles (scale bar = 20 nm). (C) UV-vis spectroscopy wavelength shift was seen after coating of GNPs with mPEG (‘mPEG-GNPs’) or PEG-COOH (‘COOH-GNPs’). (D) DLS measurements demonstrated an increase in the hydrodynamic diameter following coating of GNPs with mPEG or PEG-COOH. (E) Zeta potential measurements. Surface charge of bare GNPs, GNPs PEGylated either with PEG-COOH or mPEG, and Ab-GNPs types synthesized *via* covalent binding or adsorption. Shown are representative characterizations of GNPs with 20 mg cetuximab. Results presented as mean (of 3 samples) ± SD. mPEG-GNPs: mPEG coated GNPs; COOH-GNPs: PEG-COOH coated GNPs.

We designed Ab-GNP platforms with either covalently conjugated or adsorbed antibodies. To this end, 20 nm GNPs were synthesized and coated with either PEG-COOH or mPEG. Cetuximab, a clinically used EGFR inhibitor, served as a representative model antibody for Ab-GNP investigations. To create covalently conjugated Ab-GNPs, GNPs coated entirely with PEG-COOH were conjugated with antibodies *via* EDC-NHS coupling between the carboxyl groups of the PEG coating and the antibody amine groups, using either 1 mg, 10 mg or 20 mg antibodies in the reaction. For adsorbed Ab-GNPs, antibodies (1 mg, 10 mg or 20 mg) were incubated overnight at room temperature with either the 100% PEG-COOH-coated GNPs or the 100% mPEG-coated GNPs (mPEG lacks functional groups but has the capacity to adsorb biomolecules). Fig. 1A depicts the Ab-GNP synthesis processes. Ab-GNPs were characterized using TEM, UV-vis spectroscopy, dynamic light scattering and zeta potential. TEM showed that 20 nm GNPs with uniform size and spherical shape were obtained (Fig. 1B). Coating efficacy was confirmed by the wavelength shift in UV-vis (Fig. 1C and S1†), the increase in hydrodynamic diameter as compared to bare GNPs (Fig. 1D), and reduction in negative zeta potential (Fig. 1E). Notably, PEG-COOH coated GNPs had more negative charge than neutral mPEG-coated GNPs, consistent with the non-ionic nature of mPEG. Moreover, for all GNP types, the zeta potential measurements showed an increase in negative charge after the antibody binding step, regardless of the binding method employed, confirming the successful binding of antibodies upon GNPs (Fig. 1E). Further, binding stability of the Ab-GNPs was examined in PBS and cell culture medium, showing only negligible (0–1%) release of antibodies from both adsorbed and covalently bound GNPs after 24 h of incubation (Fig. S2A†). Incubation of PEG-COOH coated Ab-GNPs with solutions that either disrupt non-covalent bonds (saline, Tween 20, or MgCl_2_) or disrupt covalent bonds (DTT) confirmed successful covalent binding for these particles (Fig. S2B†).

### Impact of binding strategies on the antibody binding yield

**Fig. 2:**
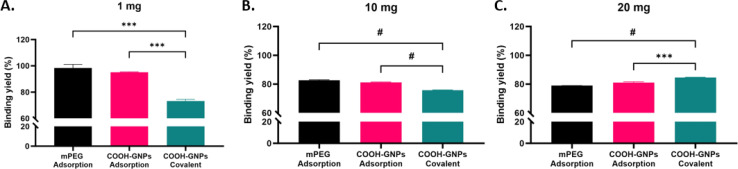
Ab-GNP binding yield for adsorption *vs.* covalent binding. GNPs coated with either mPEG or PEG-COOH (COOH-GNPs) and reacted with (A) 1 mg, (B) 10 mg, or (C) 20 mg antibodies, *via* either covalent or adsorption strategies, were assessed for binding yield. Results are presented as mean (*n* = 3 samples) ± SD. (one-way ANOVA; ***p < 0.001; #*p* < 0.0001).

We first assessed the antibody binding yields generated by either covalent binding or adsorption approaches. BCA assay was used to quantify antibodies in the reaction supernatant, and the yield of binding to GNPs was then calculated (as detailed in Methods). We observed high binding yields of ∼70–100% for all Ab-GNPs types (at 1, 10 and 20 mg initial antibody mass; Fig. 2A–C). The yield for adsorption (upon both mPEG- and PEG-COOH-coated Ab-GNPs) was significantly higher than that of covalent binding at 1 mg and 10 mg initial antibody mass, but this trend reversed at 20 mg, and the yield was significantly higher for covalent binding as compared to both adsorbed Ab-GNP types (*p* < 0.001–0.0001, Fig. 2A–C). It is notable that when examining trends within each PEG-COOH coated GNP type, for covalently-bound Ab-GNPs the binding yield increased from ∼70 to >80% when the initial antibody mass was increased (*p* < 0.05 for 1 mg and 10 mg *vs.* 20 mg), while for adsorbed Ab-GNPs the yield decreased from 95% to ∼80% when initial antibody mass increased (*p* < 0.01 for 1 mg *vs.* 10 and 20 mg). Taken together, these results indicate that the adsorption strategy was more effective in facilitating attachment of antibodies onto GNPs at lower initial antibody mass, while covalent binding was more effective for binding at higher initial antibody mass.

### Covalent binding is a superior strategy for enhanced Ab-GNP functionality

**Fig. 3:**
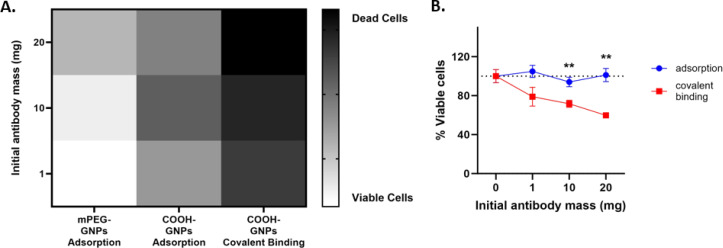
Anti-cancer cell functionality of Ab-GNPs. Each Ab-GNP type was incubated with A431 cells (72 h) and % viability was then assessed. (A) Representative heatmap showing the effect of different Ab-GNPs on cell viability. Each box in the heatmap represents a distinct type of Ab-GNP, categorized by the binding method, PEG coating type, and initial antibody mass in the synthesis reaction. Darker boxes indicate a higher number of dead cells. (B) % Viability of cells after treatment with PEG-COOH coated GNPs (with no bound antibodies (0 mg initial antibody mass point)), or with PEG-COOH-coated covalently bound or adsorbed Ab-GNPs prepared with either 1 mg, 10 mg, or 20 mg initial antibody mass. Results normalized to cells treated with PEG-COOH-coated GNPs without antibodies. Results presented as mean (*n* = 3 samples) ± SEM. Multiple *t*-test; **p < 0.01.

Next, effect of the synthesis approach on Ab-GNP cancer cell killing abilities was investigated. Human squamous cell carcinoma A431 cells were treated (72 h, 37 °C) with Ab-GNPs of each type (50 μg mL^−1^ final gold concentration for each), and cell viability was assessed using methylene blue assay. A heatmap (Fig. 3A) indicated that covalently bound Ab-GNPs had a stronger cell killing effect than the adsorbed Ab-GNP types, at 1, 10 and 20 mg initial antibody mass. We further found that PEG-COOH Ab-GNPs synthesized *via* covalent binding significantly decreased cell viability, as the initial antibody mass (and, in accordance, the binding yield) increased, as opposed to PEG-COOH Ab-GNPs synthesized using adsorption that did not affect viability (covalent Ab GNPs, 1 mg and 10 mg antibodies *vs.* 20 mg antibodies, *p* < 0.01; and covalent *vs.* adsorbed Ab-GNPs, *p* < 0.01 at 10 and 20 mg; Fig. 3B). mPEG coated Ab-GNPs, and GNPs coated only with COOH-PEG or mPEG (without antibodies), did not affect cell viability. Treatment of A431 cells with free antibodies (at doses of 180 nM and 320 nM; equivalent to doses of bound antibodies upon Ab-GNPs (at 10 mg and 20 mg initial antibody mass)) led to a decrease in cell viability at a level similar to covalently bound Ab-GNPs at the highest dose (Fig. S3†). It is notable that in the literature, a similar level of decrease in cancer cell viability and proliferation is reached following treatment with free cetuximab, at doses up to the molar range.^46,53,54^ Covalently bound Ab-GNPs did not affect viability of 3T3 fibroblast cells, similar to free antibodies (Fig. S4†), indicating the specificity of Ab-GNPs to the cancer cells, and no cytotoxicity towards non-cancerous cells with low EGFR expression.

These results clearly demonstrate that covalent binding of antibodies upon GNPs retains antibody functionality, even when using a lower initial antibody mass (for which the adsorption method had led to a better binding yield). This may be explained by the conjecture that the orientation of antibodies can be improved using the covalent binding strategy,^55^ while the adsorption approach can lead to binding of antibodies in a less favorable orientation upon the GNPs,^55,56^ or to their embedment within the PEG mesh,^57^ resulting in impaired anti-cancer cell activity.

As covalently bound PEG-COOH Ab-GNPs demonstrated a significant effect on cancer cell viability, we next evaluated the effect of varying PEG-COOH coating ratios on Ab-GNP functionality. GNPs (20 nm) were coated with either 15%, 50%, or 100% PEG-COOH (for the first two types, the remaining coating was completed by mPEG coating), and antibodies (either 1 mg, 10 mg or 20 mg in initial reaction) were then covalently bound to the particles. These different Ab-GNP types were then incubated with A431 cells (72 h; 50 μg mL^−1^ final gold concentration for each particle type), and viability was measured by methylene blue assay.

**Fig. 4:**
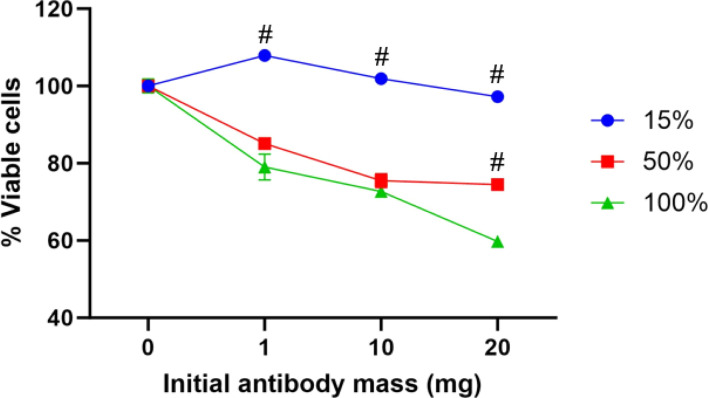
COOH-PEGs percentage effect on Ab-GNPs properties. GNPs were PEGylated with different percentages of COOH-PEGs followed by viability test. A431 cell viability were incubated (72 h) with Ab-GNPs with increasing PEG-COOH ratios. #*p* < 0.0001 for 15% *vs.* 50% and 100% PEG-COOH coated Ab-GNPs; and for 50% *vs.* 100% coated Ab-GNPs with 20 mg initial antibodies. *p* < 0.01 for 1 mg *vs.* 10 mg and 20 mg antibodies, within the 50% and 100% coated Ab-GNP groups (two-way ANOVA with Sidak's correction for multiple comparisons).

We found that Ab-GNPs with 50% and 100% PEG-COOH coating and increasing initial antibody mass significantly decreased cell viability, as compared to Ab-GNPs with 15% PEG-COOH coating that had no effect on viability (*p* < 0001; Fig. 4). Moreover, for both 50% and 100% PEG-COOH coated Ab-GNPs, the increase in initial antibody mass led to increased cell death (*p* < 0.01). These results indicate that the PEG-COOH ratio can affect the cancer killing abilities of Ab-GNPs. This may be due to the fact that a lower percentage of PEG-COOH coating entails a higher percentage of mPEG coating upon the same GNP, which leads to more adsorbed – and less covalently bound – antibodies upon each GNP. In addition, the increased amount of available carboxylic groups that were present upon GNPs may have led to more opportunities for favorably oriented binding of the antibodies,^58^ and thus to improved anti-cancer functionality.

## Discussion

Antibody-based therapies can be greatly enhanced by nanotechnology. GNP platforms for antibodies, in particular, are gaining recognition as promising systems for targeted therapy of tumors.^21,44,59^ Development of successful and effective targeted GNP-based delivery systems relies on understanding the dynamics of GNP-antibody interactions and importantly, how different synthesis approaches affect the functionality of GNP-bound antibodies. In the present study, we show that the choice of the antibody binding method can significantly impact the functionality of Ab-GNP platforms. We found that the covalent binding of antibodies generated Ab-GNPs with enhanced cancer cell killing functionality as compared to the adsorption approach. Moreover, a higher initial antibody mass in the reaction, and a higher ratio of PEG-COOH coating for covalently bound Ab-GNPs, led to a higher rate of cell death. Our findings thus highlight the importance of synthesis strategies while also taking into consideration the initial antibody mass and linker compositions, when designing antibody-GNP platforms.

The differences in functionality between the synthesized Ab-GNP types could be explained by the effect of each binding strategy on the antibody binding strength, or on antibody orientation. As for binding strength, the adsorption strategy relies mainly on electrostatic interactions between the antibodies and GNPs.^31^ The charge distribution upon antibodies can affect both the strength and stability of its interactions with the GNPs and its surface coatings. COOH-PEG coated GNPs have relatively negative charge, which can enable adsorption *via* electrostatic interactions with the antibodies' positively charged regions. Nanoparticles such as mPEG-GNPs have neutral surface charge, and as they lack inherent surface charge the antibody binding to GNPs primarily relies on non-specific interactions, including hydrophobic forces, van der Waals interactions, and steric effects.^37,38,60^ As the adsorption of the antibodies is based on weak and unstable bonds, this synthesis approach can result in desorption of the antibodies from the GNP. Thus, while adsorption led to a higher quantity of antibody binding (at lower initial antibody masses), the possibility of the antibodies' desorption from the GNPs over time may have led to diminished tumor cell killing efficacy. Covalent binding, on the other hand, provides a stronger and more long-lasting attachment of antibodies to the GNPs, preventing dissociation of antibodies from the platform.^32,39^ Regarding antibody orientation upon GNPs, this is an important factor for Ab-GNP functionality, due to the need to preserve the integrity and functionality of the antibodies' Fab region after binding, and thus ensure effective tumor antigen targeting. Oliveira *et al.* have demonstrated that the physical adsorption method yields less than 40% of favorably oriented antibodies (*i.e.*, bound *via* the Fc region and not Fab)^58^ upon GNPs. They postulated that the Fab regions are involved in binding to GNPs, which can render the antibodies' active site inaccessible, thereby reducing their antigen recognition efficiency.^58^ This may explain our findings showing low functionality of Ab-GNPs synthesized *via* adsorption. Covalent binding, however, can promote more favorably oriented antibodies upon GNPs,^15,31,56^ and thus likely enable better antigen–antibody recognition. Moreover, the covalent bond can prevent the re-orientation of antibodies on the nanoparticles during experimental or biological processes.^32,39^ We note that it is challenging to measure the exact orientation in which an antibody is attached to PEG-COOH, and the exact number of carboxyl groups bound; yet, our results show higher cancer cell killing efficacy with an increasing % PEG-COOH coating, indicating increased antibody binding yield or improved orientation. Indeed, we assume that not all of the bound antibodies on Ab-GNPs are in the active state, but nonetheless, our results also show a direct relationship between the amount of antibodies and anti-cancer functionality, therefore we assume that proper orientation was, at least largely, achieved. Future investigations should be conducted to study this issue. Taken together, our results indicate that the covalently bound Ab-GNPs can offer stabler and more precise interactions with the tumor cell surface antigens, which may have led to their enhanced cancer cell killing ability.

Our present findings are supported by previous studies which analyzed the efficiency of Ab-GNPs, generated either by physical adsorption or covalent conjugation, to serve for biosensing applications.^58^ In the abovementioned study by Oliveira *et al.*, Ab-GNPs were assessed as a biosensor for the detection of 17β-estradiol,^58^ showing that the covalent binding strategy of an anti-17β estradiol antibody led to increased sensitivity in detection, likely due to improved orientation of the antibodies on the spheric GNP surface as compared to the adsorption approach.^58^ Another study compared nonspecific adsorption and protein A-mediated immobilization of anti-horseradish peroxidase antibodies upon GNPs for biosensing.^61^ The study found that although the antibody surface coverage was lower for protein A-mediated binding than for direct adsorption, the antibodies bound through protein A nevertheless had higher enzymatic activity in solution than the antibodies that were directly adsorbed to GNPs. While previous research has assessed differences between Ab-GNP binding strategies, our study provides evidence of the superiority of covalent binding in terms of the cancer cell-killing functionality.

Beyond the insights into the functionality of covalently bound Ab-GNPs, our research builds upon previous work by our group^41,42,44^ and by others,^13^ demonstrating the value and profound effects of such Ab-GNPs both *in vitro* and *in vivo*. For instance, we have shown that GNPs conjugated to cetuximab serve as targeted radiosensitizer agents in different *in vivo* cancer models, including head and neck cancer^41^ and orthotopic glioblastoma^42^ models. Moreover, we demonstrated that GNPs conjugated to anti-programmed death ligand 1 (anti-PD-L1) antibody for immune checkpoint blockade prevented tumor growth with only a fifth of the standard dosage of clinical care, and also allowed prediction of therapeutic response.^59^ Thus, the integration of therapeutic antibodies, together with the diagnostic as well as radiosensitizing abilities of GNPs, creates theranostic nanoplatforms with potential for versatile and multifaceted applications in biomedicine.

The study of adsorption and covalent binding strategies focusing on parameters of initial antibody mass and PEG ratio, while advantageous, nevertheless warrants exploration of the effect of other parameters that may affect binding, such as GNP size and length of PEG linkers, and of additional coatings, such as glucose for enhanced tumor cell uptake^62,63^ or therapeutic drugs for creating antibody–drug–GNP conjugates. It is worth mention that a previous study that examined the effect of pH on adsorption of immunoglobulin G onto GNPs, showed that it is efficiently adsorbed throughout a range of concentrations and at both acidic and alkaline pH values.^64^ It is further notable that our research focused on a specific model antibody, and that different antibody types used for binding to GNPs could yield different results. Interestingly, cellular internalization mechanisms of Ab-GNPs have been shown in previous studies to involve energy-dependent receptor-mediated endocytosis.^65–68^ Specifically, cetuximab-bound GNPs display enhanced EGFR-mediated endocytosis, which was suggested to involve both clathrin-dependent and clathrin-independent pathways.^67^ These issues, in the context of covalent *vs.* adsorbed Ab-GNPs, should be addressed in future studies. Importantly, optimizing Ab-GNPs synthesis methodologies by systematic *in vivo* assessments should further enhance translational knowledge.

## Conclusions

The present study underscores the importance of selecting appropriate synthesis strategies to enhance the functionality of Ab-GNP platforms. Investigation of the different parameters affecting Ab-GNP functionality has the potential to unlock a wide array of applications in cancer diagnostics and therapeutics. The present research can promote the development of more efficient and precise GNP-based drug delivery systems with improved targeting efficiency and bioactivity for cancer treatment.

## Data availability

All data generated or analyzed during this study are included in this manuscript.

## Author contributions

Conceptualization, A. A. and R. P.; data curation, A. A.; formal analysis, A. A. and C. T.-A.; investigation, A. A.; methodology, A. A., C. T.-A. and M. M.; project administration, M. M.; supervision, R. P.; writing—original draft, A. A.; writing—review and editing, T. S. and R. P. All authors have read and agreed to the published version of the manuscript.

## Conflicts of interest

The authors declare no conflicts of interest.

## Supplementary Material

NA-OLF-D4NA00134F-s001
