# Supplementary material for: Impact of synthesis methods on the functionality of antibody-conjugated gold nanoparticles for targeted therapy
Source: Nanoscale Adv. 2024 Sep 4;6(21):5420–9. doi: 10.1039/d4na00134f (PMC11372556; doi:10.1039/d4na00134f)
Supplement: NA-006-D4NA00134F-s001 [file NA-006-D4NA00134F-s001.pdf]

Supplementary Information (SI) for Nanoscale Advances.
This journal is © The Royal Society of Chemistry 2024

Supplementary Information

Impact of Synthesis Methods on the Functionality of Antibody-Conjugated Gold Nanoparticles for Targeted Therapy

Adi Anaki, Chen Tzror-Azankot, Menachem Motiei, Tamar Sadan, Rachela Popovtzer*

Faculty of Engineering and the Institute of Nanotechnology and Advanced Materials, Bar-Ilan University, Ramat Gan 5290002, Israel

*Corresponding Author

Email: rachela.popovtzer@biu.ac.il

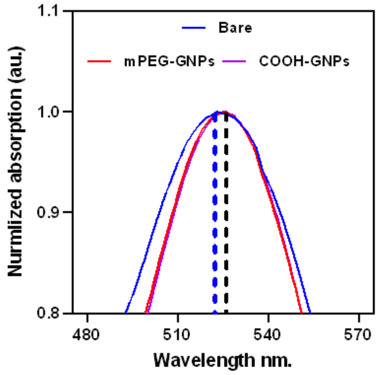

Figure S1: PEGylated-GNPs UV-visible spectrum. UV-visible spectrum of GNPs before coating ('bare GNPs'), and after coating with mPEG ('mPEG-GNPs') or PEG-COOH ('COOH-GNPs'). The UV-vis spectrum shifted after coating with the different PEG types, confirming successful coating.

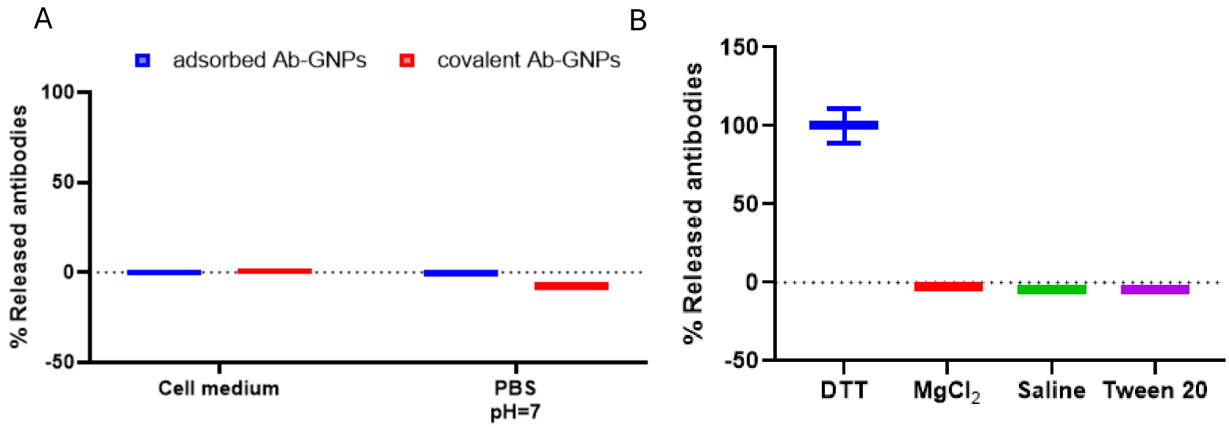

Figure S2: Stability of Ab-GNPs and confirmation of covalent binding. (A) Adsorbed Ab-GNPs or covalently bound Ab-GNPs incubated with PBS or cell culture medium (DMEM supplemented with 10% fetal bovine serum). Results are normalized to the absorbance of PEGylated-GNPs w/o antibodies. Negligible (0-1%) release of antibodies was seen from either adsorbed or covalently bound Ab-GNPs after 24 hrs of incubation with PBS or cell medium, indicating their stability. (B) Covalently-bound Ab-GNPs (100% PEG-COOH coating, 1 mg initial antibody mass) were incubated for 24h with different solutions that disrupt non-covalent bonds: Saline, Tween 20, and $MgCl_2$. The particles were also incubated with DTT, that breaks covalent bonds. Antibody release did not occur following incubation with saline, Tween 20, or $MgCl_2$, while incubation with DTT led to high release of antibodies from the GNPs. This indicates an effective process of covalent binding of the antibodies to the GNPs.

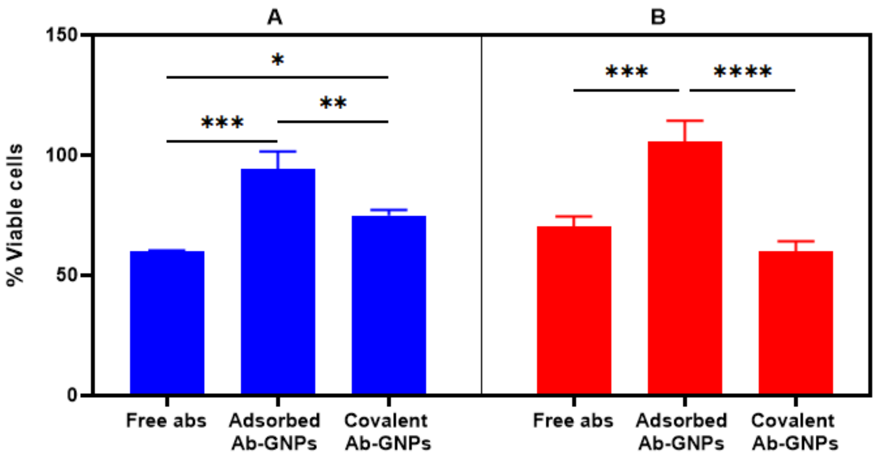

Figure S3: Equivalent effect of covalent Ab-GNPs and free antibodies on cell viability. (A) %Viable cells after treatment (72 hrs, 37°C) with free cetuximab, adsorbed Ab-GNPs, or covalent Ab-GNPs; for each group, antibodies (free/bound) at a concentration of 180 nM (both Ab-GNP types synthesized with 10 mg initial antibody mass). (B) %Viable cells after treatment (72 hrs, 37°C) with free antibody, adsorbed Ab-GNPs or covalent Ab-GNPs; for each group, antibodies (free/bound) at a concentration of 320 nM (both Ab-GNP types synthesized with 20 mg initial antibody mass). *p<0.05, **p<0.01, ***p<0.001, one way ANOVA.

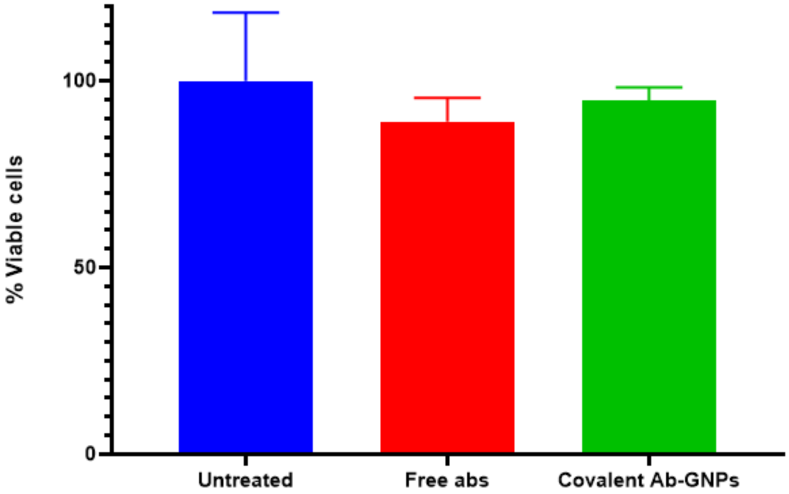

Figure S4: 3T3 fibroblast cell viability after treatment with Ab-GNPs. 3T3 cells were incubated (72 hrs, 37°C) with either PEG-COOH coated Ab-GNPs (320 nM bound antibodies; 20 mg initial antibody mass) or free antibodies (320 nM). Both treatments had no significant effect on the viability of 3T3 cells as compared to untreated cells.
